# Attitudes and perceived knowledge of health professionals on the food labelling reform in Israel

**DOI:** 10.1017/S1368980023000447

**Published:** 2023-07

**Authors:** Sharon Furman-Assaf, Moran Accos-Carmel, Tatyana Kolobov, Moran Blaychfeld-Magnazi, Ronit Endevelt, Orly Tamir

**Affiliations:** 1 The Pesach Segal Israeli Center for Diabetes Research and Policy, Sheba Medical Center, Ramat Gan, Israel; 2 Ministry of Health, Jerusalem, Israel; 3 Faculty of Social Welfare & Health Sciences, University of Haifa, Haifa, Israel

**Keywords:** Food labelling, Nutrition, Health professionals, Eating habits, Policy

## Abstract

**Objectives:**

To assess the attitudes and perceived knowledge of health professionals regarding the food product judgemental-labelling reform that began in January 2020 in Israel.

**Design:**

Cross-sectional survey.

**Settings:**

An online survey among health professionals working in the Israeli health system.

**Participants:**

456 participants (118 physicians, 207 nurses, 131 nutritionists).

**Results:**

Most respondents (89·9 %) were women, 36 % had over 20 years of professional experience. All nutritionists, 96·6 % of physicians and 94·7 % of nurses reported hearing about the reform, and most (88·9 % of nurses, 76·3 % of physicians and 75·6 % of nutritionists) claimed supporting the reform to a great or very great extent. Most respondents believe they should discuss issues related to healthy eating with their patients (91·8 % of nurses, 94·9 % of physicians and all nutritionists), but only about half (47·5 % of physicians and 57·0 % of nurses) reported that they have sufficient knowledge in this field, particularly about food labelling. Almost two-thirds of nutritionists (60·3 %) reported instructing patients to change their food intake according to labelling *v*. 40·1 % and 34·7 % of nurses and physicians, respectively. Only some respondents felt that they could influence their patients’ nutrition habits. Most participants believe that additional regulatory measures should also be used to promote healthy nutrition.

**Conclusions:**

There is a gap between the desire of physicians and nurses to provide nutritional guidance to the public and their actual knowledge about the labels’ meaning as well as their competencies in providing nutrition counselling. When formulating a reform, policymakers should provide clear guidelines about the expectations of implementing it in therapeutic practice.

In recent years, the rates of overweight and obese individuals in Israel have increased. The latest surveys reported that 17·9 % of children aged 6–7 years^(1)^, 30·2 % of adolescents aged 12 years^(2)^ and 58·5 % of adults^(1)^ are overweight or obese. Among the leading causes of obesity are dietary habits that include the consumption of high-sugar foods (e.g. sugary drinks, snacks and sweets) and other processed and ultra-processed foods^(3)^. The increasing obesity rates have led the Israeli Ministry of Health to initiate and promote a pioneering food labelling reform mandating manufacturers to place red warning labels indicating ‘high in [nutrient of concern]’ on the front packaging of food and beverages with added sugars, saturated fats or sodium that are above-established cut-offs^(4)^. In addition, products whose composition is in line with the Ministry of Health’s national nutrition recommendations for a healthy population (which are based on the Mediterranean diet) are marked with a voluntary green label^(5)^. The labels are intended to reflect to the public that red labels mean unhealthy food and green labels mean healthy food. The reform was implemented in two stages, with the first stage in January 2020 and the second stage in January 2021, with nutrient cut-offs becoming increasingly stricter over the implementation period^(6)^.

Food nutrition labels, defined as tags, marks, pictorials or other descriptive text, written or printed, attached to a pre-packaged food container, are important tools for informing consumers about the nutritional properties of the food and its health claims^(7)^. There are many types of front-of-pack labelling (FOPL), including labels with a nutrient declaration that can vary in design (i.e. shape, size, colour), in the message they convey, in their public health objective (prohibitive, prescriptive or both) and in their focus on specific nutrients. Some FOPL provide the percentage of energy and nutrients in relation to a standard value and/or to the portion available for consumption, while others provide ratings of the content (low, medium, high) of specific nutrients^(8)^.

Israel was the second country in the world to lead a binding judicial reform in food labelling. The first country to do so was Chile, which implemented the first national system of mandatory front-of-packaging warning labels for sugar-sweetened beverages and energy-dense, non-essential foods (the Law of Food Labelling and Advertising) in 2016^(9)^. A study that examined the impact of product labelling on shopping behaviour in Chile has shown that the reform significantly reduced the purchase of sweetened cereals by 11 % and the purchase of juices and soft drinks by 23·8 %, but the purchase of sweets, chocolate products and cookies did not significantly decrease^(10)^. In addition to the decrease in purchasing, it was found that after the initial application of the Chilean law of food labelling and advertising, manufacturers reformulated some groups of packaged foods and beverages. As a result, the number of sugary products (cereals, sweet baked goods and sweet spreads) decreased from 80 % to 60 %, and the number of ‘sodium-rich’ products (in salty spreads, cheeses, ready-to-eat meals, soups and sausages) decreased from 74 % to 27 %^(11)^. Additionally, Chilean authorities reported that more than 1500 products were modified to be offered with no or fewer labels^(12)^. In a survey among 1067 adults of different socio-economic groups in Chile, 93 % reported that they recognise the warning labels, 31 % preferred to buy products with fewer marks and 10 % reported not buying products with warning labels at all^(13)^. Mothers from various socio-economic groups also reported awareness of the law but indicated changing their purchase habits only when buying new products^(14)^. It is also important to note that beyond the labelling of food products, Chile has implemented taxation of sugar-sweetened beverages^(15)^ and two laws for the prevention of advertising harmful food to children, including a ban on advertising on the packaging^(16)^.

According to the Diffusion of Innovations Theory, opinion leaders who adopt a new technology (early adopters) accelerate and expand its application in the general society^(17,18)^. The public often regards health professionals as a source of authority and knowledge. Therefore, health professionals can serve as conduits for the dissemination of knowledge to the public and for changing the public’s behaviour and their approach to health reforms, particularly in areas of promoting a healthy diet and healthy lifestyle, which may have a far-reaching impact on the general population^(19)^.

Many studies have reported significant knowledge gaps among physicians, interns and nurses in understanding nutrition^(20–23)^. Such gaps in knowledge on issues related to healthy eating and awareness of food labelling policies among health professionals mean that the goals of the food labelling reform may not reach their full potential.

To understand the setting for the study, we must first provide some background about the Israeli healthcare system, and specifically about its primary care services. Israel has a national health insurance system that provides universal coverage to all citizens and permanent residents of Israel. Each individual can freely choose from four competing, not-for-profit health funds, which provide their members with access to a statutory benefits package. The public healthcare system is financed by general taxes and an earmarked payroll tax (health tax). These funds administer and provide primary and secondary care, finance and sometimes provide hospitalisation services. Municipalities are in charge of some preventive care and public health services, such as maternal and child health centres (‘Milk Drop’) and some even run hospitals. The members in each health plan choose their primary and specialist community-based physicians from physicians affiliated with the health plan^(24–26)^. Most physicians and nurses work in the public health system. Some physicians also work concomitantly in the private health system. Within the public health system, health fund members are entitled to receive nutrition counselling by certified nutritionists for various reasons and medical diagnoses related to nutrition and/or weight. Payment is by quarter regardless of the number of sessions with the nutritionist and is mostly covered by the members’ health insurance; the deductibles are approximately ILS30 per quarter (as of November 2022). This payment is significantly lower than private nutrition counselling.

Education about healthy nutrition by primary healthcare professionals may contribute to changing patients’ lifestyle habits and improving their health. However, the training of medical students in Israel does not include courses on nutrition. They sometimes learn about nutrition in relation to specific diseases, such as diabetes. Nursing training sometimes includes courses on nutrition, depending on the program. Therefore, knowledge on nutrition among physicians and nurses is not uniform or extensive. Nutritionists’ training involves an academic programme (3–4 years) in nutritional sciences at an institution that is recognised by the Ministry of Health. This is followed by a 6-month internship, at least 4 months of which are at a general hospital, and the rest of the internship is done at primary care clinics, health bureaus, chronic hospitalisation institutions, the Ministry of Education, the Israel Defense Force or at sports nutrition centres. To receive a license from the Ministry of Health to practice nutrition, a governmental exam must also be passed.

To understand if the labelling reform has achieved its goals and to identify gaps that could help policymakers understand the changes that should be further undertaken to improve public health, we examined the perceived knowledge and attitudes of Israeli health professionals on the food labelling reform.

## Methods

### Setting and participants

This cross-sectional study was an online survey conducted among physicians, nurses and nutritionists working in the Israeli public healthcare system. The study was approved by Sheba Medical Center’s ethics committee (approval number 7037–20-SMC, dated 5 May 2020). The requirement to sign an informed consent was waived. All participants were assured anonymity. Respondents were included in the analysis if they were physicians, nurses or nutritionists who currently work in the Israeli health system. Other healthcare professionals were excluded from the analysis.

### Sample

We used a layer sample methodology. According to the study design, the overall sample size, in line with a 95 % confidence interval level and a sampling error of 5 %, had to be equal to or greater than 383 subjects. Because of the expected difficulty in recruiting health professionals to participate in the survey and based on response rates in previous surveys^(24)^, we assumed that in the current study, the response rate would not be high, so we set a conservative target for the sample size of about 400 subjects. Based on the ratio of physicians, nurses and nutritionists in Israel and while making a correction that would allow statistical reference, we increased the number of nutritionists in the sample and reduced the number included in the other two strata. The final sample was calculated to include 100 physicians, 200 nurses and 100 nutritionists.

### Questionnaire

The questionnaire was constructed by the study team and was reviewed by an expert panel. A pilot questionnaire was distributed among ten professionals (from the three target sectors – physicians, nurses and nutritionists) and was modified following their feedback. The final questionnaire (online Supplementary data) comprised eighteen questions and included: demographic and professional information (age, sex, profession, seniority in the profession, workplace), knowledge questions about sugar, saturated fat and sodium consumption in Israel, perceptions about the food labelling reform and its goals, the degree of support for the reform, the impact of the reform on food consumption habits, attitudes regarding regulatory interventions to improve nutrition among the public, mentoring patients regarding the reform and perceiving the caregiver’s ability to do so.

### Data collection

The questionnaire was disseminated in ‘Google Forms’. Prior to the dissemination of the electronic questionnaire, its usability and technical functionality were tested by the study team. Data were collected from June to August 2020. Participants self-completed the questionnaire by clicking a link that was sent to them by professional group leaders (The Israeli Dietetic Association, the Head Nurse at the Ministry of Health, Family Physicians) via email, WhatsApp groups or Facebook groups. The information entered by participants was captured in a Microsoft Excel file.

### Statistical analysis

The data were analysed using IBM SPSS 25.

Categorical variables were summarised by number and frequency, and continuous variables were summarised by mean and sd. Research variables and professional groups were compared using chi-squared test (χ^2^) and one-way analysis of variance (ANOVA). *P* value < 0·05 was considered statistically significant.

## Results

A total of 456 participants (118 physicians, 207 nurses and 131 nutritionists) completed the survey. The respondents’ demographic characteristics are summarised in Table 1. Most respondents (89·9 %) were women, and more than half were aged 35–54 years. About one-third of the participants (33·8 %) reported that their main place of work is a hospital, 42·5 % reported working mainly in a health maintenance organisation and the rest stated that they work in the Ministry of Health, a private clinic, a maternal and child health clinic (‘milk drop’) and the Israel Defense Force. Most respondents reported working in the public healthcare system. Only 1·9 % of nurses and 2·5 % of physicians reported working in the private healthcare sector compared to 16·8 % of nutritionists. Thirty-six percent of the respondents had more than 20 years of professional experience.

**Table 1:** Participant characteristics

	Participants *n* 456	
	*n*	%
Profession		
Physicians	118	25·9
Nurses	207	45·4
Nutritionists	131	28·7
Gender		
Men	46	10·1
Women	410	89·9
Age, years		
18–34	91	20·0
35–54	255	55·9
> 55	110	23·4
Main place of work		
Hospital	154	33·8
Health maintenance organisation	194	42·5
Other^*^	108	23·7
Healthcare sector		
Public	394	86·4
Private	62	13·6
Years of experience		
< 10	7	1·5
10–20	285	62·5
> 20	164	36·0

* Other = Ministry of Health, private clinic, maternal and child health clinic (‘milk drop’) and the Israel Defense Force.

Table 2 shows the attitudes of different health professionals toward promoting healthy eating during their routine work. Most health professionals thought it was their job to talk about healthy nutrition issues with their patients, but only about half of physicians and nurses (47·5 % and 57·0 %, respectively) reported that they have enough knowledge in this field. About two-thirds of nutritionists (64·1 %) and only about one-third of physicians (35·5 %) and nurses (29·4 %) thought that the majority of their patients would try to change their lifestyles if they recommended it to them (*P* < 0·01 between the nutritionists and the other professionals).

**Table 2:** Attitudes to promotion of health nutrition habits by profession

	Nurses	Physicians	Nutritionists	χ2
	%	%	%	
Is it your job to raise issues of healthy eating with your patients?	91·8	94·9	100	11·3*,†
Do you think you have sufficient knowledge to guide your patients on issues of healthy eating?	57·0	47·5	99·2	90·8*,†
By virtue of your role, it is your job to promote an issue of healthy eating to your patients, but you do not have sufficient knowledge for this	38·4	50·0	0·8	42·3*,‡
My patients will try to change their lifestyle if I recommend it	29·4	35·5	64·1	61·7*,†

* *P* < 0·01,

† Significant difference between nutritionists and the two other groups.

‡ Significant among all groups.

Half of physicians and over one-third of nurses felt that they are not sufficiently proficient in nutrition issues despite their desire to raise these issues with their patients (χ2 = 42·3, *P* < 0·01).

The Ministry of Health’s food product labelling reform was well-known to health professionals. All nutritionists, 96·6 % of physicians and 94·7 % of nurses reported being exposed to the reform, with a significant difference between nutritionists and other professionals (χ2 = 7·1, *P* < 0·05). Most respondents (88·9 % of nurses, 76·3 % of physicians and 75·6 % of nutritionists) claimed that they supported the reform to a great or very great extent. Interestingly, among the three professions, nutritionists supported the reform the least (χ^2^ = 20·8, *P* < 0·01).

Participants’ perceptions about the meaning of food product labelling were not uniform. As can be seen in Table 3, there was no consensus among professions regarding the meaning of the red and green labels. Regarding the red label: 6·9 % of the nutritionists, 15·3 % of physicians and 23·7 % of nurses indicated that all products marked with a red label are unhealthy, and 39·7 % of nutritionists, 39 % of physicians and 25·6 % of nurses thought that only some of the foods marked with a red label are unhealthy, with the answers of the nutritionists being significantly different from those of other professionals (χ2 = 26·5, *P* < 0·01). About a third (35·9 %) of nutritionists and a quarter of physicians (24·6 %) and nurses (25·6 %) responded that all products marked with a green label are healthy. In addition, 22·1 % of nutritionists, 34·7 % of physicians and 35·3 % of nurses thought that only some of the foods marked with a green label are healthy. The answers of nutritionists were significantly different from those of other professionals (χ2 = 16·7, *P* < 0·01).

**Table 3:** Attitudes to red and green labels

	Nurses	Physicians	Nutritionists	χ2
	%	%	%	
All the products marked in red are unhealthy	23·7	15·3	6·9	26·5*,†
**Most of the products marked in red are unhealthy**	49·3	42·4	53·4	
Some of the products marked in red are unhealthy	25·6	39·0	39·7	
Don’t know	1·4	3·4	0·0	
**All the products marked in green are healthy**	25·6	24·6	35·9	16·7*,†
Most of the products marked in green are healthy	36·2	33·9	41·2	
Some of the products marked in green are healthy	35·3	34·7	22·1	
Don’t know	2·9	6·8	0·8	

* *P* < 0·01,

† Significant difference between nutritionists and the two other groups.

Bold indicates the correct answer according to the Ministry of Health.

The participants were also asked if they instructed their patients to change their food consumption in accordance with the food labelling (Table 4). There was a significant difference between the answers of nutritionists and those of other professionals (χ2 = 50·5, *P* < 0·01): 60·3 % of nutritionists reported instructing patients to change their food intake according to the food labels, compared with 40·1 % of nurses and 34·7 % of physicians. In addition, 11·5 % of nutritionists, 8·5 % of physicians and 4·3 % of nurses claimed that they did not instruct patients about the labels because they believe that the symbols are inaccurate. Close to half of physicians (48·3 %) and nurses (45·4 %) and 14·5 % of nutritionists reported that it is not their job to give explanations about food labelling.

When asked about changes to their own food consumption habits following the food labelling reform, close to a third of physicians (29·7 %), about half of nurses (52·7 %) and about one-fifth of nutritionists (18·3 %) reported that they have changed their habits as a result of the reform to a large or very large extent. When asked if they thought there was a change in the buying habits of the public, about 47·3 % of nutritionists thought that the buying habits of the public had changed to a great or very great extent compared to 21·2 % of physicians and 31·4 % of nurses. A significant difference was found between the answers of nutritionists and those of other professionals (*P* < 0·01).

When asked about the measures the state should take to improve public health in the context of nutrition, the most prominent recommendations were subsidising healthy food products (88·2 %), healthy eating education (84·9 %) and restricting the sale of unhealthy products (72·4 %). No significant differences were found among health professions regarding these three measures (Table 5).

**Table 4:** Instructing patients to change consumption in accordance with the reform

	Nurses	Physicians	Nutritionists	χ2
	%	%	%	
I instruct my patients to change consumption according to the reform	40·1	34·7	60·3	50·5*,†
I instruct my patients to change consumption according to the red labelling only	7·2	5·1	4·6	
I instruct my patients to change consumption according to the green labelling only	2·9	3·4	9·2	
I do not instruct my patients about the labelling reform and I make it clear to them that the symbols are not accurate	4·3	8·5	11·5	
I do not instruct my patients about the labelling reform and it is not my job	45·4	48·3	14·5	

* *P* < 0·01;

† Significant difference between nutritionists and the two other groups.

**Table 5:** The methods that the state should use to improve public health with an emphasis on healthy nutrition

	Nurses	Physicians	Nutritionists
	%	%	%
Subsidizing healthy food products	86·5	88·1	90·8
Education for healthy eating habits	84·5	85·6	84·7
Restriction on the sale of unhealthy products	69·1	70·3	79·4
Restriction on advertising unhealthy products	3·9	0·0	0·0
Imposing a tax on manufacturers on the production of unhealthy products	1·9	1·7	0·8
Imposing a tax on the consumer on unhealthy products	1·0	1·7	1·5
Subsidizing costs of gyms	1·0	0·8	2·3
The state should not intervene	1·4	1·7	0·0

## Discussion

Our results show that 6 months after the initiation of the food labelling reform, most health professionals were exposed to it, and most also supported it. These findings are similar to those obtained in relation to the general public about 2 months after the implementation of the reform (unpublished data).

The Ministry of Health does not provide specific guidelines about the role of physicians and nurses in providing nutritional guidance to patients and encouraging the use of FOPL. Nevertheless, the vast majority of respondents felt it was their job to talk to their patients about issues related to sensible nutrition, but only some felt they could influence patients’ habits and only half (excluding nutritionists) indicated that they had sufficient knowledge in the field.

Previous studies have found that physicians reported a lack of skills, knowledge and confidence in introducing lifestyle modifications to their patients’ management plans^(27–31)^. Nurse practitioners reported that formal nutrition education was lacking in their graduate school programme^(32)^. As already mentioned above, physicians and nurses in Israel receive only little training in nutrition during their professional studies. In a synthesis of surveys conducted in the UK, 28 % of doctors preferred to get specialist advice rather than address nutrition themselves^(30)^. Furthermore, adequate training in counselling was a predictor of strong self-efficacy for counselling in diet^(27)^. In a study that evaluated the personal health behaviours of physicians-in-training and attending physicians in association with patient-related lifestyle counselling, only 10·8 % of trainees and 17·3 % of attending physicians reported high self-efficacy for changing patients’ diet-related behaviours^(27)^. A qualitative study conducted among nurse practitioners working in primary care in the USA has shown that although nurse practitioners understand the importance of providing nutrition counselling in primary care practice and provide it in some capacity, the continuance of nutrition counselling is limited by barriers such as lack of time and lack of continuity of care with certain clinic structures. Nurse practitioners who obtained certifications to enhance their nutrition knowledge and counselling skills did not need to refer patients for nutrition counselling^(32)^. Therefore, it seems that physicians and nurses must receive more training in nutrition for them to feel more confident in providing counselling on such subjects.

Less than half of physicians and nurses provided the correct answer that most of the products labelled in red are unhealthy, and a quarter of the respondents from these two professions provided the correct answer that all products labelled in green are healthy. Moreover, only half of the nutritionists (53 %) provided the correct answer about the red label, and only about one-third (35 %) provided the correct answer about the green label. These findings indicate that perhaps the Ministry of Health did not communicate well enough the meaning of the food labels to health professionals, despite a mass media campaign that included informative articles on the importance of red (negative) and green (positive) FOPL, letters sent to healthcare professionals to explain the reform and some webinars provided to nutritionists. Additionally, the expectations of the Ministry of Health regarding the reform were not communicated to health professionals. It should be emphasised that the COVID-19 pandemic outbreak in Israel (March 2020) started soon after the reform came into effect, probably diverting public and medical professionals’ attention to other issues. The findings also suggest that nutritionists may not agree with the meaning of the labels as several healthy products received a red label due to their high content of saturated fat (e.g. mixed nuts) or sodium (e.g. cottage cheese). As a result, some nutritionists may have perceived that healthy foods are sometimes mislabelled as unhealthy. Additional factors may have also affected public opinion on unhealthy food products, including that of health professionals who have less knowledge of nutrition. For example, some manufacturers have placed additional labels next to the red label, such as ‘sugar from fruit only’, which may confuse the public and weaken the intended message to some extent. The literature lacks studies on how health professionals perceive FOPL. In a study that evaluated the attitudes, self-perceived proficiency and knowledge related to clinical nutrition among 66 internal medicine interns, most respondents felt particularly inadequate in their ability to counsel patients on serving sizes and food labels; only one-third were confident in their ability to analyse food labels^(23)^.

As for the achievements of the reform, about one-third of physicians and about half of nurses reported that they have changed their food consumption habits to a great or very great extent as a result of the reform. The direction of this partial effect is not known; it is possible that some of the physicians and nurses already had healthy habits before the start of the reform and did not change their own habits. Alternatively, some of them may have had unhealthy habits, and they improved them following the reform. The effect of the food labelling reform was lower among nutritionists, probably due to their extensive knowledge and better nutritional habits. Better dietary habits are associated with a higher frequency of dietary counselling for patients^(27,29)^.

One-fifth of physicians and one-third of nurses estimated that there has been a change in the purchasing habits of the public following the reform. In contrast, among nutritionists, nearly half estimated that there has been a positive change in public habits, probably due to their deeper acquaintance with patients requiring nutritional counselling. In this regard, we note that according to findings from a survey we have conducted among the public, about one-third (32 %) of respondents reported that they try to reduce food products with a red label, a quarter reported buying more products marked in green and a quarter (28 %) reported not being affected by the reform (unpublished data). In a study conducted in Chile, where the first national system of mandatory front-of-packaging warning labels for sugar-sweetened beverages and energy-dense, non-essential foods was implemented^(9)^, declines in overall purchases of calories and nutrients of concern were observed due to reductions in calories, sugar, sodium and saturated fat from unhealthy ‘high-in’ food and beverage purchases^(33)^. A similar effect was observed among Canadians who participated in an experimental marketplace study^(34)^. Notably, in Chile, the decrease in the purchase of unhealthy ‘high-in’ food and beverages were partly offset by an increase in purchases of food and beverages that were ‘not-high-in’ these nutrients of concern, resulting in a non-significant change in overall calories purchased^(33)^.

The prevailing position among the respondents was that it was important to implement additional regulatory tools, including subsidising healthy food products, restricting sales of unhealthy products and providing education on healthy eating. Similar positions were found among the public^(35)^. The World Health Organization has suggested using economic tools, such as targeted taxes and/or subsidies, to discourage the consumption of less healthy options, and to improve the consumption of healthier food products by increasing accessibility, availability and affordability^(36,37)^. It has been suggested that several regulatory tools should be applied together to achieve the desired results^(33,38,39)^. In Mexico, a 1 peso per litre excise tax on sugar-sweetened beverages and an 8 % tax on non-essential energy-dense food have led to an average reduction of 7·6 % in purchases of taxed beverages over the first 2 years of implementation, but purchases of untaxed beverages increased by 2·1 % and water purchases also increased^(40)^. A modelling study conducted in New Zealand showed that a 20 % subsidy on fruit and vegetables and an 8 % tax on saturated fat, sugar, salt and high-processed food might lead to health expenditure savings across the remaining lifespan per capita from $492 (334–694) for the junk food tax to $2164 (1472–3122) for the sugar tax^(41)^. In a study conducted in Australia, restricting the promotion of unhealthy foods, specifically items contributing most to free sugar sales, reduced the sales of free sugar, targeted beverages, sugar-sweetened beverages and confectionary^(42)^.

The limitations of the study include its cross-sectional design, which only allowed us to see the effect of the food labelling reform at a single point in time – about 6 months after its implementation. In addition, the data were collected during the first 6 months of the COVID-19 pandemic; therefore, it is possible that health professionals were concerned with other health issues. Although we wanted an optimal representation of health professionals who work in primary care settings and specifically within the public health system, since this was a convenience sample, we could not control the demographic representation of the study populations. Self-reported attitudes within each healthcare profession may not only reflect a personal, subjective opinion but may also differ by the roles and responsibilities of the individual healthcare professional within his or her medical establishment or organisation. For example, primary care physicians may have different views and perceptions than physicians or nurses working in hospitals. Furthermore, we did not ask the participants how they have dealt with their knowledge gap in nutrition (i.e. whether they referred patients to dietitians, ignored nutrition or provided general advice). In addition, selection bias may have impacted the results, as responders may be those who have concerns about nutrition and healthy eating habits, and therefore the impact of the reform on health professionals may be overestimated. Due to the length of the questionnaire, it was not possible to ask in a targeted manner about each of the labels and types of labelled products. It is possible that answering these questions would have made it possible to better understand certain aspects of knowledge gaps.

## Conclusions, policy implications and recommendations

Although most respondents to this survey have heard of the food labelling reform and support it, there seems to be a gap between physician and nurses’ desire to provide nutritional guidance to the public and their actual knowledge about the meaning of the labels and their competencies in providing counselling in nutrition. Therefore, the finding of this study might help to raise awareness among policymakers about this gap. They suggest that when formulating a reform, policymakers should provide clear guidelines about the expectations of implementing it in therapeutic practice. Physicians and nurses are not expected to replace the role of nutritionists. Time constraints, institutional roles and responsibilities may prevent them from discussing healthy nutrition and raising awareness of FOPL with every patient who comes to the clinic, but they are expected to discuss it (and refer the patient to a nutritionist) in cases where treatment of the disease involves improvement of nutrition, for example, in diabetes or hyperlipidaemia. As the current nutrition education of physicians and nurses is lacking, they may not feel confident enough to even raise this issue with their patients. Therefore, to increase their nutrition competencies, physicians and nurses should receive nutrition education courses during their professional training. This education should include information, knowledge and tools on nutrition and food labelling, particularly regarding food products whose labelling is considered controversial. In addition, information about FOPL should be disseminated to practicing physicians and nurses in primary care through professional training, courses, seminars, staff meetings, etc. In Israel, the National Program for Quality Indicators in Community Healthcare collects data on seventy indicators, including data on preventive, diagnostic and rehabilitative care provided in the community and furnishes information to policymakers and the public^(1)^. The addition of quality indicators in nutrition may help in bridging the gap between the desired policy and actual practice.

While nutritionists do not require additional nutritional education, they too should be provided with more information about FOPL, as some of them showed a lack of knowledge about the red labels or did not agree with the labelling. This finding also points to a gap between the policy formulated by the public health establishment and the knowledge of clinical nutritionists who work with patients. Therefore, the data obtained in this study can help raise awareness among policymakers about the importance of providing the appropriate information to nutritionists prior to implementing such a reform. The knowledge of nutritionists should not be taken as obvious. They too must be educated about the reform.

We assume that if health professionals had perfect knowledge and positive attitudes with respect to the FOPL, it would help raise awareness about healthy eating habits among the general population; however, as this policy is new in Israel, it should be the subject of a further study.

Greater awareness about the importance of nutrition in the prevention and treatment of disease could achieve a new paradigm for improving interdisciplinary, team-based provision of healthcare.
